# Supplementary material for: The genome sequence of the Coltsfoot Fly, Acidia cognata (Wiedemann, 1817) (Diptera: Tephritidae)
Source: Wellcome Open Res. 2026 Oct 2;11:785. [Version 1] doi: 10.12688/wellcomeopenres.28013.1 (PMC13722853; doi:10.12688/wellcomeopenres.28013.1)
Supplement: Peer review history

# Reviewer report: Reviewer response for version 1

**DOI:** 10.21956/wellcomeopenres.30818.r173567

**Published:** 2026-10-30

**Authors:** Marco Salvemini

**Affiliations:** 1 Università degli Studi di Napoli "Federico II", Naples, Italy

McCulloch and colleagues present an interesting and potentially valuable manuscript: a high-quality, haplotype-resolved, chromosome-level assembly of the Coltsfoot Fly, Acidia cognata (Diptera: Tephritidae), generated within the Darwin Tree of Life project. The methods follow the well-established Tree of Life pipeline, the data are publicly available, and the resource will be valuable for comparative genomics of Tephritidae, a family that includes several major agricultural pests. I agree with indexing after minor revisions to improve clarity.

 Minor Revisions

 Use of a single specimen for HiFi and Hi-C. The specimen is a single small adult male (wing length ~5–7 mm). It was also partly dissected for DNA barcoding, and “whole organism” tissue is reported for both the HiFi and the Hi-C libraries. The authors should explain more clearly how the tissue was divided between the HiFi and Hi-C workflows: how much tissue went to each, and whether the 2.0 mg used for HMW DNA extraction was a portion of the specimen or its entirety. This would help readers working on similarly small insects plan their own sampling.

 Identification and phasing of the sex chromosomes. The criteria used to identify the X and Y chromosomes are not described (read coverage? blob plot showing a scaffold at roughly half coverage? Hi-C contact patterns?). Please clarify.

 Phenology: In the background, the authors state that the species is univoltine, with adults emerging in May and June. However, the specimen was collected as an adult on 1 August 2022. The authors may wish to comment on this, or to extend the reported flight period.

 Mention whether an ortholog of the tephritid male-determining gene MoY (Meccariello et al., 2019, Science) can be detected in the assembled Y sequences.

 Figure 3: The Y chromosome is not labeled (only a dot appears on the top and left axes, and OZ325065.1 is missing). The legend could also mention the conspicuous inter-chromosomal contacts between pericentromeric regions.

 Typos: “et al..” (double full stop) in the citations in the main text of Twyford et al., Gries et al., and Rhie et al.; “completenessis” (missing space).

Are the datasets clearly presented in a usable and accessible format, and the assembly and annotation available in an appropriate subject-specific repository?

Yes

Are sufficient details of the sequencing and extraction, software used, and materials provided to allow replication by others?

Partly

Are the rationale for sequencing the genome and the species significance clearly described?

Yes

Are the protocols appropriate and is the work technically sound?

Yes

Reviewer Expertise:

Genomics, Developmental genetics, Bioinformatics, Vector biology

I confirm that I have read this submission and believe that I have an appropriate level of expertise to confirm that it is of an acceptable scientific standard.

# Reviewer report: Reviewer response for version 1

**Published:** 2026-10-02

**Authors:** Automated Benchmarking

**Affiliations:** 1 Wellcome Sanger Institute, Hinxton, UK

As this article is a Genome Note, Automated Benchmarking has been used to determine whether the reported assembly passes four defined checks established by the Earth BioGenome Project: the raw and assembled data are publicly available, the Earth BioGenome Project three-part metric is met, the k-mer completeness exceeds 90%, and the BUSCO score passes the stated threshold. For Acidia cognata (idAciCogn1), all four checks passed. Data available: pass. ENA study PRJEB77505, raw-read accessions ERR13362659 and ERR13363438, and assembly accessions GCA_976940545.1 and GCA_976940535.1 are public. Earth BioGenome Project three-part metric: pass. Haplotype 1 is reported as 6.C.Q65, meeting the 6.C.Q40 reference standard. k-mer completeness: pass. The combined haplotype assemblies have 95.24% k-mer completeness, exceeding the Earth BioGenome Project target of 90%. BUSCO: pass. Haplotype 1, which is proposed as the reference assembly for this species, has 99.1% complete BUSCOs (98.1% single-copy and 1.0% duplicated). BUSCO software version: 6.0.0; lineage dataset: diptera_odb10. It meets the target of greater than 90% complete and single-copy BUSCOs. This decision uses values reported in the Genome Note and public accession records.

Reviewer Expertise:

NA

I confirm that I have read this submission and believe that I have an appropriate level of expertise to confirm that it is of an acceptable scientific standard.
